# Microstructured Sensors: The Nexus of Innovation and Functionality

**DOI:** 10.3390/mi15121523

**Published:** 2024-12-21

**Authors:** Mingliang Li

**Affiliations:** Department of Chemistry, The University of Hong Kong, Hong Kong 999077, China; liml@hku.hk

In an era of rapid technological evolution, the demand for functional sensors that can keep up with the pace is more pressing than ever. Sensors have become ubiquitous in our daily lives, serving as the bedrock for various applications ranging from environmental monitoring and healthcare to security and defense [1]. However, the increasing complexity of these applications necessitates sensors that are not just functional, but also highly sensitive, accurate, and efficient. This is where microstructured sensors come into play.

Microstructured sensors, with their intricate designs and small-scale structures, offer a level of precision and efficiency that is unparalleled by their conventional counterparts [2,3]. Their minute size does not compromise their functionality but rather enhances it, allowing for the detection of subtle changes that might otherwise go unnoticed. The microstructure design is at the heart of these sensors’ enhanced performance. By manipulating the sensor’s microstructure, researchers can control its physical properties, thereby fine-tuning its sensitivity and responsiveness. This precise control over the sensor’s performance is a game-changer, heralding a new era of high-performance sensing.

In this Special Issue of *Micromachines*, we delve into the exciting world of microstructured sensors, shedding light on their design, fabrication, and application. The Special Issue includes five papers, each exploring a different facet of microstructured sensors, ranging from inkjet-printed resistive temperature detectors and flexible three-dimensional force tactile sensors to advanced biosensors for detecting waterborne bacteria and cytokines, as well as an explosive sensor based on self-assembled monolayers.

Zorman et al. examined the impact of microstructure on the performance of resistive temperature detectors (RTDs) that were inkjet-printed using Ag [4]. The researchers specifically probed the temperature coefficient of resistance (TCR) and the sensitivity of the sensors created using these inks. They discovered that the ink solvent played a substantial role in shaping the microstructure and that there was a significant negative correlation between porosity and TCR. However, they found that the sensor’s sensitivity was not heavily impacted by its microstructure, but was more influenced by the resistance of the RTD.

Zhang et al. introduced a flexible three-dimensional force tactile sensor with a sandwich structure, which is constructed from a polyethylene–carbon composite material, also known as velostat [5]. The sensor demonstrated high sensitivity across a wide measurement range, being capable of effectively detecting both shear and normal forces. Additionally, the sensor exhibited superior repeatability, stability, and efficient response and recovery rates. To demonstrate its practical application, a glove-like sensor array was created, which was capable of conducting grasping tests. The forces exerted during these tests could be monitored and displayed in real-time on a personal computer.

Yue et al. developed a Surface Plasmon Resonance (SPR) sensor of high phase sensitivity, employing a Ag–TiO_2_–Franckeite–WS_2_ hybrid structure [6]. This sensor was designed using an advanced seeker optimization algorithm (ISOA) with the aim of identifying waterborne bacteria in environmental monitoring. By employing the ISOA, the sensor’s performance and computational efficiency saw significant improvements compared to the conventional layer-by-layer technique and the standard SOA method. This advancement is expected to enable the sensor to detect a broader spectrum of bacteria with increased effectiveness.

Tu et al. introduced an all-dielectric metamaterial terahertz biosensor, notable for its high Q factor [7]. They designed and fabricated a structure that leverages the effect of electromagnetically induced transparency to carry out a Mie resonance for the terahertz response. The biosensor demonstrated a low detection limit for the cytokine interleukin 2 (IL-2) and a linear response, suggesting its significant potential in cytokine detection in serum samples, as well as its potential application in the clinical detection of cytokine release syndrome.

Li et al. prepared the TPE-PA-8 molecule, utilizing the classic aggregation-induced emission (AIE) property of 1,1,2,2-tetraphenylethene (TPE), [8] for the development of self-assembled monolayers (SAMs) [9,10] targeting the detection of trace nitroaromatic compound (NAC) explosives [11]. Due to the high signal-to-noise ratio of SAMs, these SAMs, which were assembled on Al_2_O_3_-deposited fiber films, exhibit a remarkable detection performance, with detection limits of 0.68 ppm for trinitrotoluene. This research offers a potential pathway for the design and construction of flexible sensors that are capable of the high-performance, user-friendly detection of trace NACs. 

In essence, this Special Issue underscores the importance of microstructure design in enhancing the performance of functional devices. It highlights the urgent need for sophisticated sensors that can meet the challenges of today’s complex applications. As we delve into the realm of microstructured sensors, we are not just exploring a new technology, we are pioneering the future of sensing.
